# Supplementary material for: Global sensitivity analysis for stochastic models of infectious diseases: Re-emergence of yellow fever virus in the Americas as a case study
Source: PLoS Comput Biol. 2026 Nov 3;22(11):e1013952. doi: 10.1371/journal.pcbi.1013952 (PMC13750661; doi:10.1371/journal.pcbi.1013952)
Supplement: Supplementary file 1 [file pcbi.1013952.s001.pdf]

Supplementary Information

Nate Kornetzke[1] and Helen J. Wearing[1,2]

[1]Department of Mathematics and Statistics, University of New Mexico, Albuquerque, New Mexico

[2]Department of Biology, University of New Mexico, Albuquerque, New Mexico

1 Reaction Rates

**Biological Event**	**State Variable Change**	**Rate**
	**Urban Host Transmission Cycle**	
Susceptible host infected by infectious mosquito, entering a latency period	$(S_h^u, E_h^u) \rightarrow (S_h^u - 1, E_h^u + 1)$	$b^u \beta_{mh} S_h^u \frac{I_m^u}{N_h^u}$
Exposed host becomes infectious	$(E_h^u, I_h^u) \rightarrow (E_h^u - 1, I_h^u + 1)$	$\epsilon_h E_h^u$
Infectious host recovers	$(I_h^u, R_h^u) \rightarrow (I_h^u - 1, R_h^u + 1)$	$\rho_h I_h^u$
	**Urban Mosquito Transmission Cycle**	

Biological Description	State Variable Change	Rate
Susceptible mosquito infected by an infectious host, entering a latency period	$(S_m^u, E_m^u) \rightarrow (S_m^u - 1, E_m^u + 1)$	$b^u \beta_{hm} S_m^u \frac{I_h^u}{N_h^u}$
Exposed mosquito becomes infectious	$(E_m^u, I_m^u) \rightarrow (E_m^u - 1, I_m^u + 1)$	$\epsilon_m E_m^u$
**Urban Mosquito Demographics**		
Mosquito recruitment	$(S_m^u, N_m^u) \rightarrow (S_m^u + 1, N_m^u + 1)$	$\Theta^u N_m^u$
Susceptible mosquito death	$(S_m^u, N_m^u) \rightarrow (S_m^u - 1, N_m^u - 1)$	$\frac{\Theta^u}{K^u} N_m^u S_m^u$
Exposed mosquito death	$(E_m^u, N_m^u) \rightarrow (E_m^u - 1, N_m^u - 1)$	$\frac{\Theta^u}{K^u} N_m^u E_m^u$
Infectious mosquito death	$(I_m^u, N_m^u) \rightarrow (I_m^u - 1, N_m^u - 1)$	$\frac{\Theta^u}{K^u} N_m^u I_m^u$
**Urban to Peri-urban Travel**		
Susceptible host movement from the urban cycle to the peri-urban cycle	$(S_h^u, N_h^u, S_h^p, N_h^p) \rightarrow (S_h^u - 1, N_h^u - 1, S_h^p + 1, N_h^p + 1)$	$\tau^u S_h^u$

Biological Description	State Variable Change	Rate
Exposed host movement from the urban cycle to the peri-urban cycle	$(E_h^u, N_h^u, E_h^p, N_h^p) \to (E_h^u - 1, N_h^u - 1, E_h^p + 1, N_h^p + 1)$	$\tau^u E_h^u$
Recovered host movement from the urban cycle to the peri-urban cycle	$(R_h^u, N_h^u, R_h^p, N_h^p) \to (R_h^u - 1, N_h^u - 1, R_h^p + 1, N_h^p + 1)$	$\tau^u R_h^u$
**Peri-Urban Human Host Transmission Cycle**		
Susceptible host infected by infectious mosquito, entering a latency period	$(S_h^p, E_h^p) \to (S_h^p - 1, E_h^p + 1)$	$b^p \beta_{mh} S_h^p \frac{I_m^p}{N_h^p + N_p^p}$
Exposed host becomes infectious	$(E_h^p, I_h^p) \to (E_h^p - 1, I_h^p + 1)$	$\epsilon_h E_h^p$
Infectious host recovers	$(I_h^p, R_h^p) \to (I_h^p - 1, R_h^p + 1)$	$\rho_h I_h^p$
**Peri-Urban NHP Demographics**		
NHP birth	$(S_p^p, N_p^p) \to (S_p^p + 1, N_p^p + 1)$	$\nu_p N_p^p$
NHP background mortality	$(S_p^p, N_p^p) \to (S_p^p - 1, N_p^p - 1)$	$\mu_p S_p^p$
NHP disease-induced mortality	$(R_p^p, N_p^p) \to (R_p^p - 1, N_p^p - 1)$	$\eta_p R_p^p$
**Peri-Urban NHP Host Transmission Cycle**		

**Biological Description**	**State Variable Change**	**Rate**
Susceptible host infected by infectious mosquito, entering a latency period	$(S_p^p, E_p^p) \rightarrow (S_p^p - 1, E_p^p + 1)$	$b^p \beta_{mp} S_p^p \frac{I_m^p}{N_h^p + N_p^p}$
Exposed host becomes infectious	$(E_p^p, I_p^p) \rightarrow (E_p^p - 1, I_p^p + 1)$	$\epsilon_p E_p^p$
Infectious host recovers	$(I_p^p, R_p^p) \rightarrow (I_p^p - 1, R_p^p + 1)$	$\rho_p I_p^p$
**Peri-Urban Mosquito Transmission Cycle**		
Susceptible mosquito infected by an infectious host, entering a latency period	$(S_m^p, E_m^p) \rightarrow (S_m^p - 1, E_m^p + 1)$	$b^p \beta_{hm} S_m^p \frac{I_h^p}{N_h^p + N_p^p} + b^p \beta_{pm} S_m^p \frac{I_p^p}{N_h^p + N_p^p}$
Exposed mosquito becomes infectious	$(E_m^p, I_m^p) \rightarrow (E_m^p - 1, I_m^p + 1)$	$\epsilon_m E_m^p$
**Peri-Urban Mosquito Demographics**		
Mosquito recruitment	$(S_m^p, N_m^p) \rightarrow (S_m^p + 1, N_m^p + 1)$	$\Theta^p N_m^p$
Susceptible mosquito death	$(S_m^p, N_m^p) \rightarrow (S_m^p - 1, N_m^p - 1)$	$\frac{\Theta^p}{K^p} N_m^p S_m^p$
Exposed mosquito death	$(E_m^p, N_m^p) \rightarrow (E_m^p - 1, N_m^p - 1)$	$\frac{\Theta^p}{K^p} N_m^p E_m^p$
Infectious mosquito death	$(I_m^p, N_m^p) \rightarrow (I_m^p - 1, N_m^p - 1)$	$\frac{\Theta^p}{K^p} N_m^p I_m^p$

Biological Description	State Variable Change	Rate
**Peri-urban to Urban Travel**		
Susceptible host movement from the peri-urban cycle to the urban cycle	$(S_h^p, N_h^p, S_h^u, N_h^u) \to (S_h^p - 1, N_h^p - 1, S_h^u + 1, N_h^u + 1)$	$\tau^p S_h^p$
Exposed host movement from the peri-urban cycle to the urban cycle	$(E_h^p, N_h^p, E_h^u, N_h^u) \to (E_h^p - 1, N_h^p - 1, E_h^u + 1, N_h^u + 1)$	$\tau^p E_h^p$
Recovered host movement from the peri-urban cycle to the urban cycle	$(R_h^p, N_h^p, R_h^u, N_h^u) \to (R_h^p - 1, N_h^p - 1, R_h^u + 1, N_h^u + 1)$	$\tau^p R_h^p$
**Peri-urban to Sylvatic Travel**		
Susceptible host movement from the peri-urban cycle to the urban cycle	$(S_p^p, N_p^p, S_p^s, N_p^s) \to (S_p^p - 1, N_p^p - 1, S_p^s + 1, N_p^s + 1)$	$\varphi^p S_p^p$
Exposed host movement from the peri-urban cycle to the urban cycle	$(E_p^p, N_p^p, E_p^s, N_p^s) \to (E_p^p - 1, N_p^p - 1, E_p^s + 1, N_p^s + 1)$	$\varphi^p E_p^p$
Susceptible host movement from the peri-urban cycle to the urban cycle	$(R_p^p, N_p^p, R_p^s, N_p^s) \to (R_p^p - 1, N_p^p - 1, R_p^s + 1, N_p^s + 1)$	$\varphi^p R_p^p$
**Sylvatic NHP Demographics**		
NHP birth	$(S_p^s, N_p^s) \to (S_p^s + 1, N_p^s + 1)$	$\nu_p N_p^s$

**Biological Description**	**State Variable Change**	**Rate**
NHP background mortality	$(S_p^s, N_p^s) \rightarrow (S_p^s - 1, N_p^s - 1)$	$\mu_p S_p^s$
NHP disease-induced mortality	$(R_p^s, N_p^s) \rightarrow (R_p^s - 1, N_p^s - 1)$	$\eta_p R_p^s$
	**Sylvatic NHP Host Transmission Cycle**	
Susceptible host infected by infectious mosquito, entering a latency period	$(S_p^s, E_p^s) \rightarrow (S_p^s - 1, E_p^s + 1)$	$b^s \beta_{mp} S_p^s \frac{I_m^s}{N_p^s}$
Exposed host becomes infectious	$(E_p^s, I_p^s) \rightarrow (E_p^s - 1, I_p^s + 1)$	$\epsilon_p E_p^s$
Infectious host recovers	$(I_p^s, R_p^s) \rightarrow (I_p^s - 1, R_p^s + 1)$	$\rho_p I_p^s$
	**Sylvatic Mosquito Transmission Cycle**	
Susceptible mosquito infected by an infectious host, entering a latency period	$(S_m^s, E_m^s) \rightarrow (S_m^s - 1, E_m^s + 1)$	$b^s \beta_{pm} S_m^s \frac{I_p^s}{N_p^s}$
Exposed mosquito becomes infectious	$(E_m^s, I_m^s) \rightarrow (E_m^s - 1, I_m^s + 1)$	$\epsilon_m E_m^s$
	**Sylvatic Mosquito Demographics**	
Mosquito recruitment	$(S_m^s, N_m^s) \rightarrow (S_m^s + 1, N_m^s + 1)$	$\Theta^s N_m^s$

**Biological Description**	**State Variable Change**	**Rate**
Susceptible mosquito death	$(S_m^s, N_m^s) \to (S_m^s - 1, N_m^s - 1)$	$\frac{\Theta^s}{K^s} N_m^s S_m^s$
Exposed mosquito death	$(E_m^s, N_m^s) \to (E_m^s - 1, N_m^s - 1)$	$\frac{\Theta^s}{K^s} N_m^s E_m^s$
Infectious mosquito death	$(I_m^s, N_m^s) \to (I_m^s - 1, N_m^s - 1)$	$\frac{\Theta^s}{K^s} N_m^s I_m^s$
**Sylvatic to Peri-Urban Travel**		
Susceptible host movement from the peri-urban cycle to the urban cycle	$(S_p^s, N_p^s, S_p^p, N_p^p) \to (S_p^s - 1, N_p^s - 1, S_p^p + 1, N_p^p + 1)$	$\varphi^s S_p^s$
Exposed host movement from the peri-urban cycle to the urban cycle	$(E_p^s, N_p^s, E_p^p, N_p^p) \to (E_p^s - 1, N_p^s - 1, E_p^p + 1, N_p^p + 1)$	$\varphi^s E_p^s$
Susceptible host movement from the peri-urban cycle to the urban cycle	$(R_p^s, N_p^s, R_p^p, N_p^p) \to (R_p^s - 1, N_p^s - 1, R_p^p + 1, N_p^p + 1)$	$\varphi^s R_p^s$

2 Model Simulation

Investigating how uncertainty of the parameter values affects the uncertainty of the quantities of interest requires being able to compute the distribution of the continuous-time Markov chain for a given parameter value, which is done by solving the Kolmogorov forward equations of the continuous-time Markov chain.

Solving these equations is often analytically and numerically intractable. Instead, Monte Carlo methods, such as the stochastic simulation algorithm, have been developed to infer the distribution by simulating trajectories whose events adhere to the underlying distribution. Approximation methods, known as tau-leaping, have subsequently been developed to reduce the computational cost associated with the original exact method. However, some tau-leaping methods are known to over count events, producing negative state variables when the state variables get close to zero [1]. Many methods have been developed to avoid this dramatic over counting, including the binomial tau-leaping method. Since our simulation starts with an initial condition where many state variables are close to 0, we are especially prone to this issue if we use the traditional tau-leaping approach. So, we use the binomial tau-leaping method [2].

We choose an initial state where there is no disease in the urban or peri-urban cycles, and 1 infected NHP in the sylvatic cycle. The values chosen for each cycle's host populations were based on population densities of $1 km^2$ found in the literature [3, 4, 5, 6, 7]. This time scale was chosen because it is roughly the average lifespan of a larger non-human primate, and previous modeling on arboviruses in non-human primates has implicated the importance of primate lifespan in disease incidence [8]. We list the initial starting populations below.

$$S_h^u(0) = 7800, E_h^u(0) = I_h^u(0) = R_h^u(0) = 0, \tag{1}$$

$$S_m^u(0) = 2(7800), E_m^u(0) = I_m^u(0) = 0, \tag{2}$$

$$S_h^p(0) = 300, E_h^p(0) = I_h^p(0) = R_h^p(0) = 0, \tag{3}$$

$$S_p^p(0) = 202, E_p^p(0) = I_p^p(0) = R_p^p(0) = 0, \tag{4}$$

$$S_m^p(0), = 2(300 + 202), E_m^p(0) = I_m^p(0) = 0, \tag{5}$$

$$S_p^s(0) = 385, E_p^s(0) = 0, I_p^s(0) = 1, R_p^p(0) = 0, \tag{6}$$

$$S_m^s(0) = 2(385), E_m^s(0) = I_m^s(0) = 0 \tag{7}$$

References

1. Gillespie DT. Stochastic simulation of chemical kinetics. Annual Review of Physical Chemistry. 2007; 58:35–55. DOI: 10.1146/annurev.physchem.58.032806.104637
2. Tian T and Burrage K. Binomial leap methods for simulating stochastic chemical kinetics. The Journal of Chemical Physics. 2004 Dec 1; 121:10356–64. DOI: 10.1063/1.1810475. Available from: https://doi.org/10.1063/1.1810475 [Accessed on: 2025 Aug 18]
3. Andrade AC de. Density of marmosets in highly urbanised areas and the positive effect of arboreous vegetation. Urban Ecosystems. 2022 Feb 1; 25:101–9. DOI: 10.1007/s11252-021-01131-5. Available from: https://doi.org/10.1007/s11252-021-01131-5 [Accessed on: 2025 Aug 18]
4. Hue T, Caubet M, and Moura ACdA. Howlers and marmosets in Pacatuba: an overcrowded existence in a semi-deciduous Atlantic forest fragment? Mammalia. 2017 Jul 1; 81:339–48. DOI: 10.1515/mammalia-2015-0167. Available from: https://www.degruyterbrill.com/document/doi/10.1515/mammalia-2015-0167/html?lang=en&srsltid=AfmBOoqhSmJvO5ZMJ2KWh4YWJSSn5q_AhUJ9czTOY6e0Wct5M20X0-8y [Accessed on: 2025 Aug 18]

5. Brazil BR: Rural Population — Economic Indicators — CEIC. Available from: https://www.ceicdata.com/en/brazil/population-and-urbanization-statistics/br-rural-population [Accessed on: 2025 Aug 18]

6. São Paulo (Municipality, Brazil) - Population Statistics, Charts, Map and Location. Available from: https://www.citypopulation.de/en/brazil/regiaosudeste/admin/s%C3%A3o_paulo/3550308__s%C3%A3o_paulo/ [Accessed on: 2025 Aug 18]

7. Ravetz J and Sahana M. Chapter 1 - Where is the peri-urban? A working definition and global typology. Modern Cartography Series. Ed. by Sahana M. Vol. 11. Remote Sensing and GIS in Peri-Urban Research. Academic Press, 2024 Jan 1:3–25. DOI: 10.1016/B978-0-443-15832-2.00001-0. Available from: https://www.sciencedirect.com/science/article/pii/B9780443158322000010 [Accessed on: 2025 Aug 18]

8. Althouse BM, Vasilakis N, Sall AA, Diallo M, Weaver SC, and Hanley KA. Potential for Zika Virus to Establish a Sylvatic Transmission Cycle in the Americas. PLoS Neglected Tropical Diseases. 2016 Dec 15; 10:e0005055. DOI: 10.1371/journal.pntd.0005055. Available from: https://www.ncbi.nlm.nih.gov/pmc/articles/PMC5157942/ [Accessed on: 2025 Aug 18]
