# Supplementary material for: Global sensitivity analysis for stochastic models of infectious diseases: Re-emergence of yellow fever virus in the Americas as a case study
Source: PLoS Comput Biol. 2026 Nov 3;22(11):e1013952. doi: 10.1371/journal.pcbi.1013952 (PMC13750661; doi:10.1371/journal.pcbi.1013952)
Supplement: Supplementary file 2 [file pcbi.1013952.s002.pdf]

The manuscript by Kornetzke and Wearing applies global sensitivity analysis techniques to a stochastic model of yellow fever virus transmission. I read this paper with great interest, and I commend the authors for their clear presentation of the model, the global sensitivity analysis methods, and the results. I believe this paper provides a compelling case study of GSA methods for infectious disease ecology, and the corresponding conclusions about YFV biology are interesting and useful.

I have two major comments for consideration before publication:

1. The manuscript refers to variation from two sources: the stochastic solver and parametric uncertainty. As the authors point out, stochasticity is a key consideration in emergence, and variation "from the stochastic solver" arises from real biological processes such as demographic stochasticity. Can the authors please clarify why we do not want to consider this variation in the GSA? Additional nuance in how this "variation from the stochastic solver" is described (relative to the biological context) would be useful.
2. This manuscript requires readers engage with two big conceptual topics: GSA and YFV. I wonder if there are changes to the presentation of methods and results that would improve ease and accessibility for readers. Some suggestions:
    a. The current structure jumps back and forth between the two topics, and there is a significant amount of mathematical detail provided on both. Together, I found this to be a bit burdensome. The authors may consider moving some YFV model details to a supplement, and/or introducing the YFV model and ecology after GSA since the results focus on the YFV model specifically.
    b. I suspect the GSA methods will be new to many readers (and I think this is a good thing!). To assist these less familiar readers, is it possible to add a simple illustrative example or figure to help build intuition? Or perhaps a short section after the methods are introduced to provide some baseline expectations (e.g., when do we expect Sobol indices to be zero?, what does it mean if first order and total order indices are approximately equivalent?) These points come up throughout the text, but it might be useful to have them all in one place.

Minor comments

1. Introduction: I was surprised that branching process theory wasn't mentioned. This is one of the key theoretical tools we have to understand emergence (e.g., Antia et al. 2004 https://www.nature.com/articles/nature02104 and many more)

2. Line 142-143: "The public health threat posed by emerging YFV…" It might be worth briefly mentioning somewhere the burden of YFV disease to support this claim.
3. Figure 2: Should the subscripts in the bottom panel boxes be m? (i.e., S_m, E_m, I_m?)
4. Equations (1-3): I can't find where some of these variables are introduced. What are x_i and Y?
5. Table 2: presumably, these same methods could be used with non-uniform parameter distributions?
6. Line 347: I do not know what "reduandersoncing" means, perhaps a more accessible term could be used instead?
7. Line 481-482 and following paragraph nicely summarizes the problem and motivates this work. I think it would be useful to introduce local sensitivity (as a common but inferior approach to GSA) in the Introduction as well to contextualize the GSA methods within the broader set of methods that readers may be more familiar with.
8. I noted a few typos while reading:
    a. Line 280: should "essentially" be "especially"?
    b. Line 313: extra "of"
