# Supplementary material for: Global sensitivity analysis for stochastic models of infectious diseases: Re-emergence of yellow fever virus in the Americas as a case study
Source: PLoS Comput Biol. 2026 Nov 3;22(11):e1013952. doi: 10.1371/journal.pcbi.1013952 (PMC13750661; doi:10.1371/journal.pcbi.1013952)
Supplement: Supplementary file 3 [file pcbi.1013952.s004.pdf]

Response to Reviewers' Comments

We thank both reviewers for taking the time to carefully read our manuscript and provide constructive feedback. Below, we address in detail the comments of the reviewers (repeated in bold) and summarize the changes that have been incorporated into the revised manuscript in response to their suggestions. All revision page numbers refer to the revised manuscript with highlights and striked-out text.

1 Response to Comments of Reviewer 1

1. This paper was not as expected from the title, which indicates the focus would be on yellow fever virus (YFV) emergence. However, the abstract and author summary focus on the global sensitivity analyses (GSA) with YFV as a mere comment. There needs to be a better alignment of the results of the paper and the framing of the paper.

We have changed the title to better align with the focus of the paper. We have also reorganized the layout to better align with the results and framing of the paper. In the revised manuscript, we begin with an introduction to stochasticity in infectious disease models, referencing the re-emergence of yellow fever virus in South America. Furthermore, we motivate why one would want to perform global sensitivity analysis. We then introduce Sobol indices and their computation, followed by the issues presented by stochastic solvers and how the variance deconvolution solves those problems.

2. The paper spends a significant amount of time in background and formulation of GSA but then focuses the results on local sensitivity analyses (two-at-a-time). For example, two of the three results figures are on local sensitivity. In addition, the two-at-a-time sensitivity are not mentioned prior to appearing in the results. In the discussion, there are comparisons to a one-at-a-time local sensitivity that does not appear in the paper.

Our manuscript originally used the terms one-at-a-time and local interchangeable, but we have changed the language throughout the manuscript to avoid this equivocation. Global sensitivity analysis quantifies how variation in a model's input affects variation in a model's output, while local sensitivity analysis quantifies how changes locally to a variable affect changes in that model's output, e.g. fixing a model input x_0 and

letting f be our model, local sensitivity analysis measures $\|f(x_0)-f(x_0+\epsilon)\|$ for small $\epsilon > 0$. A one-at-a-time model investigates how varying one parameter across its entire range, while holding the other parameters constant, affects a model's output, and thus, a one-at-a-time analysis is a type of global sensitivity analysis, since it looks at a parameter across its entire range and gives us the first-order effects — we have included a new figure in the revisions (Figure 1) to clarify what is meant by one-at-a-time analysis and first-order effects, as well as terms like higher-order effects.

We subsequently spend time on two-at-a-time analysis because the results of the GSA inform us which parameters affect the model output's variation the most, and thus which parameters we should focus on. The discussion highlights this dilemma when we mention that there are 268,435,455 possible parameter combinations for a model with 28 parameters. GSA allows us to rigorously pinpoint which parameters to focus on, the 6 parameters with non-trivial Sobol indices, lines 538-541.

3. The parameter choice and formulations are hard to follow. Many parameters are referred to as rates, but as the model is CTMC, they should not be rates. It's unclear if the terminology is wrong or if the formulation itself is incorrect. The parameter naming is confusing. On lines 182-183, it is stated that superscripts will refer to cycle and subscripts to the host or vector, but there are parameters that go against this convention. For example, the movement parameters (tau and phi) have subscripts that refer to cycle. This is particularly confusing as there is p for peri-urban and p for NHP.

First, we were not always precise when using the terms probability and rates in the original manuscript, and we thank the reviewer for honing in on this distinction. These are reaction rates; this naming convention follows that found in the stochastic simulation literature (e.g. "Stochastic Simulation of Chemical Kinetics" by Daniel T. Gillespie, 2007). It also appears in the definition of a CTMC in J.R. Norris's book *Markov Chains*, 2012. Consequently, the parameters appearing in the reaction rates are rates. Terminology in the manuscript has been changed to reflect this.

Second, we erroneously used subscripts for some of the parameters that we meant to use superscripts for. Travel parameters broke the superscript and subscript convention established at the beginning of the paper, the cycle is denoted in the superscript and the host or vector is denoted in the subscript. In the revised text, these were changed to adhere to that convention, e.g. τ_u is now τ^u.

4. The parameter values are unclear. Table 2 provides references, but from the caption these seem to be to point estimates, around which there are choices made for the interval. What value the citation in Table 2 refers to is unclear and how the interval for parameters is chosen is unclear. There is description that multiple host species are used but it's unclear if there are distinct population or a mix of parameters for a single population.

We have clarified the process of how the parameter ranges were determined by adding an additional paragraph, lines 415-432, which explains how the ranges were determined from the existing literature.

5. The results of the sensitivity analyses will depend on the interval of parameter chosen. If some of the parameters have much wider intervals, they will typically have bigger impacts on the variance of the output. It's stated that parameters with smaller intervals have less uncertainty (are better studied). While this may be the case, it makes the conclusions of the analysis here difficult to interpret.

Wider intervals reflect the uncertainty associated with a parameter, but this larger uncertainty does not necessarily translate into large uncertainty in the quantity of interest that we are interested in. How the variance of an input affects the variance of an output depends on the model structure and the quantity of interest. For example, in our model, the biting rates of the mosquitoes differ across large ranges, but none of the biting rates had any significant effect on the quantity of interest under investigation. Indeed, one reason for performing a global sensitivity analysis is that for many models we cannot a priori determine if a parameter's large variance will be significant or not for a quantity of interest.

6. The results on the movement parameters are hard to interpret. How do the patches change when the movement parameters are far from symmetric? For example, if tau_u is 0 (or very small) and tau_p is large, the expectation would be that individuals would accumulate in the urban patch, which could alter cumulative incidence. In general, it's unclear what to make of the heatmaps without an understanding of the underlying population size dynamics.

We have added the following sentences, lines 499 - 504, to the manuscript: "The total human population across the urban and peri-urban cycles is fixed (there are no births or deaths), so highly asymmetric movement parameters will lead to accumulation of the total human population in one patch. However, because the non-human primate populations include birth and death processes, asymmetric travel parameters for them do not imply the same population dynamics as we observe with the humans."

1.1 Minor Comments

- **-Line 123: A. aegypti should be italicized**
  - We fixed this.
- **- Line 170-171: Confusing to call peri-urban and urban landscapes in one sentence and cycles in the next.**
  - We changed the language here to refer to cycles instead of landscapes.

- **- Figure 1 caption: The description of the dashed arrows is confusing. It sounds like it refers to parameterization by key species.**
  - We clarified this sentence by indicating more clearly what the dashed arrows represent.
- **- Line 192: Do both humans and NHP have lifelong immunity to YFV?**
  - Yes, we clarified this assumption, line 324-325.
- **- Line 199: Unclear as movement is used to mean transition between classes, not movement between classes.**
  - We changed the language here to be less ambiguous by using "transition" instead of movement, reserving movement for the patch movements.
- **- Figure 2: The SEI model has subscripts h (human?) but that should be the mosquito model. This figure would be more complete and less difficult to understand with the state diagrams for human, NHP and mosquitoes separately.**
  - The diagrams were fixed to contain consistent subscripts.
- **- Figure 2 caption: It's stated that the carrying capacity K is set to twice the host size. Is this per patch? If so, how is the peri-urban patch set?**
  - This is for each patch, and we added this detail to the model description in the caption of Figure 3.
- **- Line 237-238: More clarity on what is meant by "probabilistic analogue to the logistic growth equation" would be helpful.**
  - We agree that this language is imprecise. Here, we assume logistic growth for the mosquito population, noting that the logistic growth equation is part of the CTMC, not a differential equation, line 371-372.
- **- Line 244: Something is off in this line. "... considerable levelsmodel diagram ..."**
  - This was a typo while doing edits, our apologies! This was fixed.
- **- Line 251: Do all SEIR classes move? If so, is this realistic?**
  - No, the infectious classes do not move, and this is included in the model section, line 390-391.
- **- Line 347: Something is off in this line. " ... while reduandersoncing the ..."**

  - This was another typo, our apologies again! This was fixed.

- **- Line 402: Suggest starting a new paragraph with "The parameters ..." This seems like a different discussion than the previous part of the paragraph.**

  - We created a new paragraph here, line 454.

- **- Figure 3: It would be helpful to use the same symbol in the text and figures for phi. It would also be helpful to have the definitions of the parameters accessible in the figure.**

  - We updated the figures to be consistent with the typography of phi.

- **- Line 406: The simplifying assumptions on YFV that were stated to be discussed below were not clear.**

  - We are not sure what the reviewer means here. Line 406 in the original manuscript does not refer to simplifying assumptions.

- **- Lines 408-411: The one-at-a-time analysis that is compared is not clear in the results. This could be easily directly shown to make the comparison.**

  - We have clarified how first-order and total-order indices reveal information about first-order effects (i.e. one-at-a-time) and higher-order effects by adding a new figure that explains these terms, Figure 1.

2 Response to Comments of Reviewer 2

The manuscript refers to variation from two sources: the stochastic solver and parametric uncertainty. As the authors point out, stochasticity is a key consideration in emergence, and variation "from the stochastic solver" arises from real biological processes such as demographic stochasticity. Can the authors please clarify why we do not want to consider this variation in the GSA? Additional nuance in how this "variation from the stochastic solver" is described (relative to the biological context) would be useful.

In a deterministic model, the output is a single trajectory, whereas in a stochastic model, the output is a distribution of trajectories. So, when we perform a GSA on a deterministic model, we have one quantity of interest to deal with, e.g. time of peak incidence, but when this model is stochastic, because we have a distribution of model outputs, our quantity of interest is some statistic of this distribution, e.g. mean time of peak incidence. If this distribution was directly available (i.e. we could write down the exact formula for it),

GSA of stochastic models would not present anymore hurdles than GSA of a deterministic model. However, the distributions we're interested in as quantities of interest are rarely directly available, and instead, they are inferred via a Monte Carlo sampling process, e.g. using Gillespie's algorithm to compute many trajectories and taking the mean of the time of peak incidence of these trajectories. This sampling process introduces sampling variance, i.e. it is the uncertainty of our approximation to the mean. It is this variance that we are removing, not the variance of the distribution itself.

To make this clearer, let us assume our model is the distribution of a random variable X, and our quantity of interest is the expectation $E[X]$. Furthermore, we suppose that $E[X]$ cannot be directly computed, but we can sample X easily with independent and identically distributed samples X_i. The sampled expectation of n samples, denoted $\tilde{X}$, is:

$$\tilde{X} = \frac{\sum_{i=1}^{n} X_i}{n} \tag{1}$$

By the law of large numbers, $\tilde{X} \to E[X]$ as $n \to \infty$. Our sampling process of X naturally has a variance, $Var(\tilde{X})$, and again by the law of large numbers, we have that $Var(\tilde{X}) \to 0$ as $n \to \infty$. Reducing the sampling variance gets us closer to the true expectation $E[X]$, and we emphasize that $Var(\tilde{X})$ is not the same as $Var(X)$, which is the variance of the distribution, i.e. our model. Indeed, the variance of the distribution $Var(X)$ can be approximated as usual:

$$Var(X) \approx \frac{n}{n-1}\Big(\sum_{i=1}^{n} \frac{x_i^2}{n} - \tilde{X}^2\Big) \tag{2}$$

And like our approximation to the true mean, we have that $\frac{n}{n-1}\Big(\sum_{i=1}^{n} \frac{x_i^2}{n} - \tilde{X}^2\Big) \to Var(X)$ as $n \to \infty$, so that $Var\left(\frac{n}{n-1}\Big(\sum_{i=1}^{n} \frac{x_i^2}{n} - \tilde{X}^2\Big)\right) \to 0$ as $n \to \infty$, i.e. the sampling variance goes to 0.

We have added clarification on what the sampling variance is, and how we deal with it, in the section on global sensitivity analysis, lines 187-206.

The current structure jumps back and forth between the two topics, and there is a significant amount of mathematical detail provided on both. Together, I found this to be a bit burdensome. The authors may consider moving some YFV model details to a supplement, and/or introducing the YFV model and ecology after GSA since the results focus on the YFV model specifically.

We have changed the ordering of the paper to begin with GSA and then move into YFV and the YFV model.

I suspect the GSA methods will be new to many readers (and I think this is a good thing!). To assist these less familiar readers, is it possible to add a simple illustrative example or figure

to help build intuition? Or perhaps a short section after the methods are introduced to provide some baseline expectations (e.g., when do we expect Sobol indices to be zero?, what does it mean if first order and total order indices are approximately equivalent?) These points come up throughout the text, but it might be useful to have them all in one place.

A figure, Figure 1, was added to the beginning of the paper to clarify what is meant by first-order, total-order, higher-order effects, etc.

2.1 Minor Comments

- **I was surprised that branching process theory wasn't mentioned. This is one of the key theoretical tools we have to understand emergence (e.g., Antia et al. 2004 https://www.nature.com/artic and many more)**
  - Thank you for this suggestion, we have added a reference to branching processes in the introduction, line 66-67.
- **Line 142-143: "The public health threat posed by emerging YFV..." It might be worth briefly mentioning somewhere the burden of YFV disease to support this claim**
  - We agree, and we added details here in the revised manuscript, line 272-273.
- **Figure 2: Should the subscripts in the bottom panel boxes be m? (i.e., S_m, E_m, I_m?)**
  - Yes, this was a mistake and the figure was fixed.
- **Equations (1-3): I can't find where some of these variables are introduced. What are x_i and Y?**
  - We apologize for not including what these variables are. We clarified what these variables are by introducing them explicitly in the revised manuscript, lines 154-155.
- **Table 2: presumably, these same methods could be used with non-uniform parameter distributions?**
  - Yes, the methods here are not restricted to uniform distributions, and we included why we used uniform distributions in the revised manuscript, lines 433-434.
- **Line 347: I do not know what "reduandersoncing" means, perhaps a more accessible term could be used instead?**

  - This was a typo, our apologies! This was fixed.

- **Line 481-482 and following paragraph nicely summarizes the problem and motivates this work. I think it would be useful to introduce local sensitivity (as a common but inferior approach to GSA) in the Introduction as well to contextualize the GSA methods within the broader set of methods that readers may be more familiar with.**
  - We erroneously used the term local sensitivity analysis when we meant to use one-at-a-time analysis. We clarified our language in the revised manuscript. We added a brief mention of an alternative GSA technique to Sobol indices, one that only investigates first-order effects, lines 550-551.

- **I noted a few typos while reading: a. Line 280: should "essentially" be "especially"? b. Line 313: extra "of"**
  - Thank you! We fixed these typos.
