# Supplementary material for: Global sensitivity analysis for stochastic models of infectious diseases: Re-emergence of yellow fever virus in the Americas as a case study
Source: PLoS Comput Biol. 2026 Nov 3;22(11):e1013952. doi: 10.1371/journal.pcbi.1013952 (PMC13750661; doi:10.1371/journal.pcbi.1013952)
Supplement: Supplementary file 4 [file pcbi.1013952.s005.pdf]

Reply to Reviewers

Nate Kornetzke, Helen J. Wearing

Note on Document

We thank each reviewer for taking the time to carefully read our manuscript and give constructive feedback. Each comment a reviewer made appears in bold, and our response to each appears below. Line references are made to the main document and SI with highlights and strikeouts.

1 Reviewer 1's Major Comments

In response to Reviewer 1, #2, the response indicates: "...Global sensitivity analysis quantifies how variation in a model's input affects variation in a model's output, while local sensitivity analysis quantifies how changes locally to a variable affect changes in that model's output, e.g. fixing a model input x_0 and letting f be our model, local sensitivity analysis measures $|f(x_0)-f(x_0+\epsilon)||$ for small $\epsilon > 0$. A one-at-a-time model investigates how varying one parameter across its entire range, while holding the other parameters constant, affects a model's output, and thus, a one-at-a-time analysis is a type of global sensitivity analysis, since it looks at a parameter across its entire range and gives us the first-order effects — we have included a new figure in the revisions (Figure 1) to clarify what is meant by one-at-a-time analysis and first-order effects, as well as terms like higher-order effects." This statement is in direct conflict with Lines 123-124 of the revisions: "GSA is a statistical analysis where we compute the variance of a quantity of interest (QoI) 124 given variance across all the model inputs [10]." While local sensitivity analysis does typically restrict the variation in the input parameter (or parameters) to a local region (as the response indicates), the key feature of GSA is not whether parameters are varied across their entire range, but that multiple input parameters are considered to adequately represent the entire suite of interdependencies defining the full underlying multivariate distribution. Even though it explores more than a single input, two-at-a-time analysis across the entire parameter ranges is typically not considered GSA because

it's only representing a slice of the underlying multivariate distribution. That being said, the language in the revised manuscript is sufficiently clear on what is being done and whether the two-at-a-time analysis truly falls into GSA may at the present time be an interpretation that differs here.

We agree that the language used here are in conflict with the claim that a one-at-a-time analysis is a type of GSA. We have modified these lines, lines 124, to make this consistent.

Throughout this paper, we follow the convention set in Saltelli et al. [1], where GSA refers to sensitivity methods that explore a parameter (or parameters) throughout its entire range, including some one-at-a-time methods. In particular, Morris's method is considered a GSA methodology, per Saltelli et al. [1], although this method explores the effects of perturbing an individual parameter. If we let $f(x_1, \ldots, x_n)$ be our model and $x_1, \ldots, x_n$ be our parameters, then Morris's method quantifies variation of the parameter $x_i \in [a, b]$ by taking the difference quotient

$$\frac{f(x_1, \ldots, x_i + \Delta x, \ldots, x_n) - f(x_1, \ldots, x_i, \ldots, x_n)}{\Delta x}, \tag{1}$$

where Δx is a fixed small value, and $x_i \in [a, b]$ is sampled via Latin-hyper cube sampling. This method has widely been recognized as GSA, e.g. [1, 2, 3, 4].

2. In response to Reviewer 1 #5, the response states: "Wider intervals reflect the uncertainty associated with a parameter, but this larger uncertainty does not necessarily translate into large uncertainty in the quantity of interest that we are interested in. How the variance of an input affects the variance of an output depends on the model structure and the quantity of interest. For example, in our model, the biting rates of the mosquitoes differ across large ranges, but none of the biting rates had any significant effect on the quantity of interest under investigation. Indeed, one reason for performing a global sensitivity analysis is that for many models we cannot a priori determine if a parameter's large variance will be significant or not for a quantity of interest." This makes complete sense. A parameter that is varied widely but does not impact the QoI very much is not very impactful. However, this response does not negate the original point that by restricting a priori to a narrow input region for a parameter makes it significantly less likely for a parameter to show high variance in the QoI. If a narrowly sampled parameter shows little impact, it just shows it's less important across the input region considered, not that it's objectively less important than other parameters. The description of how the parameter ranges were determined (added in the revision) is helpful but incomplete. For example, lines 400-401 states that " . . . the travel rates were determined by numerical

experiments, picking a wide range that allowed for stochastic die-off as well as epidemics." What range was initially considered? What was the threshold for an epidemic? Furthermore, what is the unit on this parameter? In fact, units for all the rates in Table 2 would help clarify.

GSA informs a modeler of a parameter's impact, assuming that the parameter belongs to a certain range. We cover this mathematically when we introduce the Sobol indices, where we assume that a parameter belongs to a certain range in lines 132 and Figure 1, and we also cover this point more broadly in the discussion in lines 561-576.

A narrowly sampled interval for a parameter is not a priori less likely to be impactful than a larger interval. The impact is due not only to the interval that the parameter is restricted to, but it is also dependent on the model's behavior as well. To make the point more clear, we illustrate this with a simple toy example. Let us suppose we have the function $f(x)$, where

$$f(x) = \begin{cases} \sin(x) & \text{if } x \geq 0 \\ 1 & \text{if } x < 0 \end{cases}. \tag{2}$$

This function has zero variation across any interval $[a, b] \subset (-\infty, 0)$, no matter how large we make it. However, for $\epsilon > 0$, f will have non-zero variation on the interval $[-\epsilon, \epsilon]$. The variation here did not have to due with the magnitude of the interval, but with the function's dynamics and where in parameter-space our interval is. The function's behavior changes at the point 0. The interval $[-\epsilon, \epsilon]$ will cover the function's behavior changing because this interval contains 0, while any interval $[a, b] \subset (-\infty, 0)$ will show zero variation, despite its larger magnitude.

With some analysis, it can be shown that the variance of our function f does not grow large and instead approaches $\frac{1}{2}$ as $\epsilon \to \infty$. Here, we have a toy model, where it is easy to identify where the function's behavior changes qualitatively and affects its variation. However, for many of the models we use in biological modeling, it is often not a priori possible to perform such analysis.

We strongly agree that the importance of where in parameter space a parameter's interval is chosen to lie is an important task of the modeler, as it relies on knowledge of the underlying biological system itself. We highlight this importance in the discussion, lines 561-576.

We have further clarified Table 2.

In response to Reviewer 1 #6, the response states: "We have added the following sentences, lines 499 - 504, to the manuscript: "The total human population across the urban and peri-urban cycles is fixed (there are no births or deaths), so highly asymmetric movement parameters will lead to accumulation of the total human population in one patch. However,

because the non-human primate populations include birth and death processes, asymmetric travel parameters for them do not imply the same population dynamics as we observe with the humans." This makes sense and helps clarify that the effective population size of patches is changing, but no descriptions of how this effects the interpretation of results has been included. For example, in Figure 5, each pair of τ^p and τ^u will result in a different effective population size in the urban patch so the fact that absolute disease incidence is higher when movement out of the urban cycle is low is not surprising. However, is the actual level of disease lower or higher? Perhaps a normalized outcome such as cumulative incidence per person would be more clear for comparison. How do the initial conditions alter this disease incidence? (In fact, the initial condition of no infection in the urban and peri-urban cycles and 1 infectious NHP in the sylvatic cycle is buried in the Supplemental information and it's a bit unclear what the total starting population sizes are and if mosquito carrying capacities are fixed or change with changing host population size.) If the host populations are started near effective population sizes but still only 1 infectious NHP, are the same disease incidences observed? Additional clarification on how the two-at-a-time results are impacted by changes in effective population size is needed to fully understand the results.

We want to thank the reviewer for their thoughtful considerations and recommendations. We have clarified how we measure cumulative incidence in the text, lines 363-367. We have abstained from adding additional QoIs, such as per-capita incidence, for the two-at-a-time analysis because other QoIs were not analyzed with the Sobol indices, and one of the goals of this paper is to explore how a GSA can inform a two-at-a-time analysis. We agree that additional QoIs and exploration of initial conditions are necessary to get a fuller picture of YFV's re-emergence. Sobol analysis of additional QoIs and their subsequent two-at-a-time analysis are non-trivial tasks, and that falls outside the scope of this work. We changed the title of the paper during the last round of edits to reflect this emphasis. However, we acknowledge what future work on YFV's re-emergence should contain, lines 582-585, 590-602.

The carrying capacity for the mosquitoes is listed in the description of Figure 3. We have added to the discussion, lines 590-602, the limitations of our current analysis regarding the initial conditions, which were held constant for the work presented here. This was done for model simplicity and computing tractability, but in lines 590-602, we discuss the importance of analyzing this in future work. We have also added what the starting populations are for the simulations in the SI, equations 1 - 7.

2 Response to Reviewer 1's minor comments

The inclusion of Figure 1 is very helpful but some additional clarity in the notation would further improve it. This could simply be some additional information in the caption. Define that A is a vector of parameters x_1 to x_n and B is the output of f. In fact, it may help to use Y instead of B is that is what is used in the text description.

We have changed Figure 1 to be more clear.

In Table 2, should μ_p, ν_p and η_p be rates? For example, "NHP disease death rate" rather than "NHP disease death"? Should β^p_{mh} be a probability? Without units this is hard to be certain of.

Thank you! We have fixed this, Table 2.

For Figures 5 and 6, how many CTMC replicates are used for the averages presented. Is it the same number as for the GSA (30 replicates)?

We performed 100 replicates; we have added this information to the figures, lines 465-467.

Figure 6. y-axis label of the top plot uses a different symbol for ϕ. x-axis label of the top plot uses a superscript for latency rate when it should be a subscript.

We have fixed this, thank you!

Lines 511-513 (apologies for giving the wrong line reference in the initial revision) refer to simplifying assumptions on YFV to be state below that are not clear.

Thank you! We have fixed this, lines 518-519.

Lines 459-460, for the two-at-a-time analysis, all other parameters are stated to be fixed but Table 2 only lists ranges of values (no point estimates). It is unclear what the fixed values used are, especially as some ranges are taken from the literature and not necessarily symmetric around a point estimate.

Thank you! We forgot this detail, and we added it, lines 466-467.

3 Response to Reviewer 2's minor comment

One very minor comment: the mention of branching processes in the introduction still implies that these must be simulated, but they are relatively tractable analytically (at least in simpler cases).

We have fixed this, thank you!

References

1. Saltelli A, Ratto M, Andres T, Campolongo F, Cariboni J, Gatelli D, Saisana M, and Tarantola S. Global Sensitivity Analysis. The Primer — Wiley Online Books. 2007 Dec 18. Available from: https://onlinelibrary.wiley.com/doi/book/10.1002/9780470725184 [Accessed on: 2025 Aug 18]

2. Pappenberger F, Beven KJ, Ratto M, and Matgen P. Multi-method global sensitivity analysis of flood inundation models. Advances in Water Resources. 2008 Jan 1; 31:1–14. DOI: 10.1016/j.advwatres.2007.04.009. Available from: https://www.sciencedirect.com/science/article/pii/S0309170807000747 [Accessed on: 2026 Aug 11]

3. Gasior KI. Determining parameter impact in systems biology models via sensitivity analysis: a comparative approach. npj Systems Biology and Applications. 2026 Jun 13. DOI: 10.1038/s41540-026-00757-5. Available from: https://www.nature.com/articles/s41540-026-00757-5 [Accessed on: 2026 Aug 11]

4. Herman JD, Kollat JB, Reed PM, and Wagener T. Technical Note: Method of Morris effectively reduces the computational demands of global sensitivity analysis for distributed watershed models. Hydrology and Earth System Sciences. 2013 Jul 24; 17:2893–903. DOI: 10.5194/hess-17-2893-2013. Available from: https://hess.copernicus.org/articles/17/2893/2013/ [Accessed on: 2026 Aug 11]
