# Supplementary material for: Global sensitivity analysis for stochastic models of infectious diseases: Re-emergence of yellow fever virus in the Americas as a case study
Source: PLoS Comput Biol. 2026 Nov 3;22(11):e1013952. doi: 10.1371/journal.pcbi.1013952 (PMC13750661; doi:10.1371/journal.pcbi.1013952)
Supplement: Supplementary file 5 [file pcbi.1013952.s006.pdf]

Reply to Reviewers

Nate Kornetzke

Reviewer #1: The authors have suitably addressed the comments. The work is an excellent contribution to applied literature on global sensitivity analysis. A few minor comments remain.

The authors have added units to Table 2 and clarified which quantities are rates and which are probabilities, which has greatly clarified the parameterization. However, η_p is still described as "NHP disease death" rather than "NHP disease death rate" in table 2. Updating this would be more consistent with the other rate parameters in the table.

This was updated, thank you for catching this!

Are mosquito carrying capacities fixed throughout a simulation or change as host population sizes change? Although it is mentioned that K is set to twice the host size in each patch in Fig. 3 caption, does it mean they are fixed at twice the initial host population size in each patch?

Mosquito carrying capacities are fixed at twice the initial host population in each patch throughout the simulation. We adjusted the text to account for this.
