# Supplementary material for: Global sensitivity analysis for stochastic models of infectious diseases: Re-emergence of yellow fever virus in the Americas as a case study
Source: PLoS Comput Biol. 2026 Nov 3;22(11):e1013952. doi: 10.1371/journal.pcbi.1013952 (PMC13750661; doi:10.1371/journal.pcbi.1013952)
Supplement: Peer review history

# Peer review: Decision Letter 0

**DOI:** 10.1371/journal.pcbi.1013952.r001

**Authors:** Chadi Saad-Roy, Benjamin Althouse

12 Mar 2026

PCOMPBIOL-D-26-00184

Quantifying Uncertainty in the Re-Emergence of Yellow Fever Virus

PLOS Computational Biology

Dear Dr. Kornetzke,

Thank you for submitting your manuscript to PLOS Computational Biology. After careful consideration, we feel that it has merit but does not fully meet PLOS Computational Biology's publication criteria as it currently stands. Therefore, we invite you to submit a revised version of the manuscript that addresses the points raised during the review process.

Please submit your revised manuscript by May 12 2026 11:59PM. If you will need more time than this to complete your revisions, please reply to this message or contact the journal office at ploscompbiol@plos.org. When you're ready to submit your revision, log on to https://www.editorialmanager.com/pcompbiol/ and select the 'Submissions Needing Revision' folder to locate your manuscript file.

Please include the following items when submitting your revised manuscript:

* A letter that responds to each point raised by the editor and reviewer(s). You should upload this letter as a separate file labeled 'Response to Reviewers'. This file does not need to include responses to formatting updates and technical items listed in the 'Journal Requirements' section below.

* A marked-up copy of your manuscript that highlights changes made to the original version. You should upload this as a separate file labeled 'Revised Manuscript with Track Changes'.

* An unmarked version of your revised paper without tracked changes. You should upload this as a separate file labeled 'Manuscript'.

If you would like to make changes to your financial disclosure, competing interests statement, or data availability statement, please make these updates within the submission form at the time of resubmission. Guidelines for resubmitting your figure files are available below the reviewer comments at the end of this letter

We look forward to receiving your revised manuscript.

Kind regards,

Chadi M. Saad-Roy

Academic Editor

PLOS Computational Biology

Benjamin Althouse

Section Editor

PLOS Computational Biology

**Journal Requirements:**

1) We ask that a manuscript source file is provided at Revision. Please upload your manuscript file as a .doc, .docx, .rtf or .tex. If you are providing a .tex file, please upload it under the item type u2018LaTeX Source Fileu2019 and leave your .pdf version as the item type u2018Manuscriptu2019.

2) Please upload all main figures as separate Figure files in .tif or .eps format. For more information about how to convert and format your figure files please see our guidelines:

https://journals.plos.org/ploscompbiol/s/figures

3) We have noticed that you have uploaded Supporting Information files, but you have not included a list of legends. Please add a full list of legends for your Supporting Information files after the references list.

**Reviewers' comments:**

Reviewer's Responses to Questions

**Comments to the Authors:**

**Please note here if the review is uploaded as an attachment.**

Reviewer #1: This paper describes sensitivity analyses to stochastic epidemic models, with a focus on a computationally efficient method for looking at global sensitivity via Sobol indices. In order to elucidate the global sensitivity method, the paper looks at a three patch, multi-species model of yellow fever virus. The paper concludes that only a few parameters are impactful for altering human host prevalence in the urban landscape and that several of these parameters only through higher order effects. Overall, the paper is well written.

Major comments:

1. This paper was not as expected from the title, which indicates the focus would be on yellow fever virus (YFV) emergence. However, the abstract and author summary focus on the global sensitivity analyses (GSA) with YFV as a mere comment. There needs to be a better alignment of the results of the paper and the framing of the paper.

2. The paper spends a significant amount of time in background and formulation of GSA but then focuses the results on local sensitivity analyses (two-at-a-time). For example, two of the three results figures are on local sensitivity. In addition, the two-at-a-time sensitivity are not mentioned prior to appearing in the results. In the discussion, there are comparisons to a one-at-a-time local sensitivity that does not appear in the paper.

3. The parameter choice and formulations are hard to follow. Many parameters are referred to as rates, but as the model is CTMC, they should not be rates. It’s unclear if the terminology is wrong or if the formulation itself is incorrect. The parameter naming is confusing. On lines 182-183, it is stated that superscripts will refer to cycle and subscripts to the host or vector, but there are parameters that go against this convention. For example, the movement parameters (tau and phi) have subscripts that refer to cycle. This is particularly confusing as there is p for peri-urban and p for NHP.

4. The parameter values are unclear. Table 2 provides references, but from the caption these seem to be to point estimates, around which there are choices made for the interval. What value the citation in Table 2 refers to is unclear and how the interval for parameters is chosen is unclear. There is description that multiple host species are used but it’s unclear if there are distinct population or a mix of parameters for a single population.

5. The results of the sensitivity analyses will depend on the interval of parameter chosen. If some of the parameters have much wider intervals, they will typically have bigger impacts on the variance of the output. It’s stated that parameters with smaller intervals have less uncertainty (are better studied). While this may be the case, it makes the conclusions of the analysis here difficult to interpret.

6. The results on the movement parameters are hard to interpret. How do the patches change when the movement parameters are far from symmetric? For example, if tau_u is 0 (or very small) and tau_p is large, the expectation would be that individuals would accumulate in the urban patch, which could alter cumulative incidence. In general, it’s unclear what to make of the heatmaps without an understanding of the underlying population size dynamics.

Minor comments:

- Line 123: A. aegypti should be italicized

- Line 170-171: Confusing to call peri-urban and urban landscapes in one sentence and cycles in the next.

- Figure 1 caption: The description of the dashed arrows is confusing. It sounds like it refers to parameterization by key species.

- Line 192: Do both humans and NHP have lifelong immunity to YFV?

- Line 199: Unclear as movement is used to mean transition between classes, not movement between classes.

- Figure 2: The SEI model has subscripts h (human?) but that should be the mosquito model. This figure would be more complete and less difficult to understand with the state diagrams for human, NHP and mosquitoes separately.

- Figure 2 caption: It’s stated that the carrying capacity K is set to twice the host size. Is this per patch? If so, how is the peri-urban patch set?

- Line 237-238: More clarity on what is meant by “probabilistic analogue to the logistic growth equation” would be helpful.

- Line 244: Something is off in this line. “… considerable levelsmodel diagram …”

- Line 251: Do all SEIR classes move? If so, is this realistic?

- Line 347: Something is off in this line. “ … while reduandersoncing the …”

- Line 402: Suggest starting a new paragraph with “The parameters …” This seems like a different discussion than the previous part of the paragraph.

- Figure 3: It would be helpful to use the same symbol in the text and figures for phi. It would also be helpful to have the definitions of the parameters accessible in the figure.

- Line 406: The simplifying assumptions on YFV that were stated to be discussed below were not clear.

- Lines 408-411: The one-at-a-time analysis that is compared is not clear in the results. This could be easily directly shown to make the comparison.

Reviewer #2: Please see attached.

**********

**Have the authors made all data and (if applicable) computational code underlying the findings in their manuscript fully available?**

The PLOS Data policy requires authors to make all data and code underlying the findings described in their manuscript fully available without restriction, with rare exception (please refer to the Data Availability Statement in the manuscript PDF file). The data and code should be provided as part of the manuscript or its supporting information, or deposited to a public repository. For example, in addition to summary statistics, the data points behind means, medians and variance measures should be available. If there are restrictions on publicly sharing data or code —e.g. participant privacy or use of data from a third party—those must be specified.

Reviewer #1: **No:**  Some code is available through Github but no README is provided so unclear if all data/code for reproducibility is present.

Reviewer #2: Yes

**********

PLOS authors have the option to publish the peer review history of their article (what does this mean?). If published, this will include your full peer review and any attached files.

If you choose “no”, your identity will remain anonymous but your review may still be made public.

**Do you want your identity to be public for this peer review?**  For information about this choice, including consent withdrawal, please see our Privacy Policy.

Reviewer #1: No

Reviewer #2: No

[NOTE: If reviewer comments were submitted as an attachment file, they will be attached to this email and accessible via the submission site. Please log into your account, locate the manuscript record, and check for the action link "View Attachments". If this link does not appear, there are no attachment files.]

**Figure resubmission:**

After uploading your figures to PLOS’s NAAS tool - https://ngplosjournals.pagemajik.ai/artanalysis, NAAS will process the files provided and display the results in the "Uploaded Files" section of the page as the processing is complete. If the uploaded figures meet our requirements (or NAAS is able to fix the files to meet our requirements), the figure will be marked as "fixed" above. If NAAS is unable to fix the files, a red "failed" label will appear above. When NAAS has confirmed that the figure files meet our requirements, please download the file via the download option, and include these NAAS processed figure files when submitting your revised manuscript.

**Reproducibility:**

?>

Attachment Submitted filename: review.pdf

# Author comment: Author response to Decision Letter 1

**DOI:** 10.1371/journal.pcbi.1013952.r002

27 May 2026

Attachment Submitted filename: response_to_reviewers.pdf

# Peer review: Decision Letter 1

**DOI:** 10.1371/journal.pcbi.1013952.r003

**Authors:** Chadi Saad-Roy, Benjamin Althouse

24 Jun 2026

Global Sensitivity Analysis for Stochastic Models of Infectious Diseases

PLOS Computational Biology

Dear Dr. Kornetzke,

Thank you for submitting your manuscript to PLOS Computational Biology. After careful consideration, we feel that it has merit but does not fully meet PLOS Computational Biology's publication criteria as it currently stands. Therefore, we invite you to submit a revised version of the manuscript that addresses the points raised during the review process.

Please submit your revised manuscript by Aug 24 2026 11:59PM. If you will need more time than this to complete your revisions, please reply to this message or contact the journal office at ploscompbiol@plos.org. When you're ready to submit your revision, log on to https://www.editorialmanager.com/pcompbiol/ and select the 'Submissions Needing Revision' folder to locate your manuscript file.

Please include the following items when submitting your revised manuscript:

* A letter that responds to each point raised by the editor and reviewer(s). You should upload this letter as a separate file labeled 'Response to Reviewers'. This file does not need to include responses to formatting updates and technical items listed in the 'Journal Requirements' section below.

* A marked-up copy of your manuscript that highlights changes made to the original version. You should upload this as a separate file labeled 'Revised Manuscript with Track Changes'.

* An unmarked version of your revised paper without tracked changes. You should upload this as a separate file labeled 'Manuscript'.

We look forward to receiving your revised manuscript.

Kind regards,

Chadi M. Saad-Roy

Academic Editor

PLOS Computational Biology

Benjamin Althouse

Section Editor

PLOS Computational Biology

**Additional Editor Comments (if provided):**

**Reviewers' comments:**

Reviewer's Responses to Questions

**Comments to the Authors:**

**Please note here if the review is uploaded as an attachment.**

Reviewer #1: The manuscript has significantly improved with revisions and represents an important contribution to the literature. It’s a nice explanation of how to apply GSA to stochastic models, with a relevant application with more than a toy biological result as a case study. However, there remain a few changes requested to fully clarify the manuscript.

1. In response to Reviewer 1, #2, the response indicates:

“…Global sensitivity analysis quantifies how variation in a model’s input affects variation in a model’s output, while local sensitivity analysis quantifies how changes locally to a variable affect changes in that model’s output, e.g. fixing a model input $x_0$ and letting $f$ be our model, local sensitivity analysis measures $||f(x_0)−f(x_0 +\epsilon)||$ for small $\epsilon>0$. A one-at-a-time model investigates how varying one parameter across its entire range, while holding the other parameters constant, affects a model’s output, and thus, a one-at-a-time analysis is a type of global sensitivity analysis, since it looks at a parameter across its entire range and gives us the first-order effects — we have included a new figure in the revisions (Figure 1) to clarify what is meant by one-at-a-time analysis and first-order effects, as well as terms like higher-order effects.”

This statement is in direct conflict with Lines 123-124 of the revisions:

“GSA is a statistical analysis where we compute the variance of a quantity of interest (QoI) 124 given variance across all the model inputs [10].”

While local sensitivity analysis does typically restrict the variation in the input parameter (or parameters) to a local region (as the response indicates), the key feature of GSA is not whether parameters are varied across their entire range, but that multiple input parameters are considered to adequately represent the entire suite of interdependencies defining the full underlying multivariate distribution.

Even though it explores more than a single input, two-at-a-time analysis across the entire parameter ranges is typically not considered GSA because it’s only representing a slice of the underlying multivariate distribution.

That being said, the language in the revised manuscript is sufficiently clear on what is being done and whether the two-at-a-time analysis truly falls into GSA may at the present time be an interpretation that differs here.

2. In response to Reviewer 1 #5, the response states:

“Wider intervals reflect the uncertainty associated with a parameter, but this larger uncertainty does not necessarily translate into large uncertainty in the quantity of interest that we are interested in. How the variance of an input affects the variance of an output depends on the model structure and the quantity of interest. For example, in our model, the biting rates of the mosquitoes differ across large ranges, but none of the biting rates had any significant effect on the quantity of interest under investigation. Indeed, one reason for performing a global sensitivity analysis is that for many models we cannot a priori determine if a parameter’s large variance will be significant or not for a quantity of interest.”

This makes complete sense. A parameter that is varied widely but does not impact the QoI very much is not very impactful. However, this response does not negate the original point that by restricting a priori to a narrow input region for a parameter makes it significantly less likely for a parameter to show high variance in the QoI. If a narrowly sampled parameter shows little impact, it just shows it’s less important across the input region considered, not that it’s objectively less important than other parameters.

The description of how the parameter ranges were determined (added in the revision) is helpful but incomplete. For example, lines 400-401 states that “ … the travel rates were determined by numerical experiments, picking a wide range that allowed for stochastic die-off as well as epidemics.” What range was initially considered? What was the threshold for an epidemic? Furthermore, what is the unit on this parameter? In fact, units for all the rates in Table 2 would help clarify.

3. In response to Reviewer 1 #6, the response states:

“We have added the following sentences, lines 499 - 504, to the manuscript: “The total human population across the urban and peri-urban cycles is fixed (there are no births or deaths), so highly asymmetric movement parameters will lead to accumulation of the total human population in one patch. However, because the non-human primate populations include birth and death processes, asymmetric travel parameters for them do not imply the same population dynamics as we observe with the humans.”

This makes sense and helps clarify that the effective population size of patches is changing, but no descriptions of how this effects the interpretation of results has been included. For example, in Figure 5, each pair of $\tau^p$ and $\tau^u$ will result in a different effective population size in the urban patch so the fact that absolute disease incidence is higher when movement out of the urban cycle is low is not surprising. However, is the actual level of disease lower or higher? Perhaps a normalized outcome such as cumulative incidence per person would be more clear for comparison. How do the initial conditions alter this disease incidence? (In fact, the initial condition of no infection in the urban and peri-urban cycles and 1 infectious NHP in the sylvatic cycle is buried in the Supplemental information and it’s a bit unclear what the total starting population sizes are and if mosquito carrying capacities are fixed or change with changing host population size.) If the host populations are started near effective population sizes but still only 1 infectious NHP, are the same disease incidences observed?

Additional clarification on how the two-at-a-time results are impacted by changes in effective population size is needed to fully understand the results.

Minor comments:

- The inclusion of Figure 1 is very helpful but some additional clarity in the notation would further improve it. This could simply be some additional information in the caption. Define that $A$ is a vector of parameters $x_1$ to $x_n$ and $B$ is the output of $f$. In fact, it may help to use $Y$ instead of $B$ is that is what is used in the text description.

- In Table 2, should $\mu_p$, $\nu_p$ and $\eta_p$ be rates? For example, “NHP disease death rate” rather than “NHP disease death”? Should $\beta_{mh}^p$ be a probability? Without units this is hard to be certain of.

- For Figures 5 and 6, how many CTMC replicates are used for the averages presented. Is it the same number as for the GSA (30 replicates)?

- Figure 6. y-axis label of the top plot uses a different symbol for $\phi$. x-axis label of the top plot uses a superscript for latency rate when it should be a subscript.

- Lines 511-513 (apologies for giving the wrong line reference in the initial revision) refer to simplifying assumptions on YFV to be state below that are not clear.

- Lines 459-460, for the two-at-a-time analysis, all other parameters are stated to be fixed but Table 2 only lists ranges of values (no point estimates). It is unclear what the fixed values used are, especially as some ranges are taken from the literature and not necessarily symmetric around a point estimate.

Reviewer #2: I thank the authors for carefully considering my suggestions; I believe the revisions have significantly improved the accessibility of the manuscript. Congratulations on a nice paper that brings an interesting new method to our field!

One very minor comment: the mention of branching processes in the introduction still implies that these must be simulated, but they are relatively tractable analytically (at least in simpler cases).

**********

**Have the authors made all data and (if applicable) computational code underlying the findings in their manuscript fully available?**

The PLOS Data policy requires authors to make all data and code underlying the findings described in their manuscript fully available without restriction, with rare exception (please refer to the Data Availability Statement in the manuscript PDF file). The data and code should be provided as part of the manuscript or its supporting information, or deposited to a public repository. For example, in addition to summary statistics, the data points behind means, medians and variance measures should be available. If there are restrictions on publicly sharing data or code —e.g. participant privacy or use of data from a third party—those must be specified.

Reviewer #1: Yes

Reviewer #2: None

**********

PLOS authors have the option to publish the peer review history of their article (what does this mean?). If published, this will include your full peer review and any attached files.

If you choose “no”, your identity will remain anonymous but your review may still be made public.

**Do you want your identity to be public for this peer review?**  For information about this choice, including consent withdrawal, please see our Privacy Policy.

Reviewer #1: No

Reviewer #2: No

[NOTE: If reviewer comments were submitted as an attachment file, they will be attached to this email and accessible via the submission site. Please log into your account, locate the manuscript record, and check for the action link "View Attachments". If this link does not appear, there are no attachment files.]

**Figure resubmission:**

**Reproducibility:**

?>

# Author comment: Author response to Decision Letter 2

**DOI:** 10.1371/journal.pcbi.1013952.r004

25 Aug 2026

Attachment Submitted filename: replies.pdf

# Peer review: Decision Letter 2

**DOI:** 10.1371/journal.pcbi.1013952.r005

**Authors:** Chadi Saad-Roy, Benjamin Althouse

23 Sep 2026

PCOMPBIOL-D-26-00184R2

Global Sensitivity Analysis for Stochastic Models of Infectious Diseases

PLOS Computational Biology

Dear Dr. Kornetzke,

Thank you for submitting your manuscript to PLOS Computational Biology. After careful consideration, we feel that it has merit but does not fully meet PLOS Computational Biology's publication criteria as it currently stands. Therefore, we invite you to submit a revised version of the manuscript that addresses the points raised during the review process.

Please submit your revised manuscript by Nov 23 2026 11:59PM. If you will need more time than this to complete your revisions, please reply to this message or contact the journal office at ploscompbiol@plos.org. When you're ready to submit your revision, log on to https://www.editorialmanager.com/pcompbiol/ and select the 'Submissions Needing Revision' folder to locate your manuscript file.

Please include the following items when submitting your revised manuscript:

* A letter that responds to each point raised by the editor and reviewer(s). You should upload this letter as a separate file labeled 'Response to Reviewers'. This file does not need to include responses to formatting updates and technical items listed in the 'Journal Requirements' section below.

* A marked-up copy of your manuscript that highlights changes made to the original version. You should upload this as a separate file labeled 'Revised Manuscript with Track Changes'.

* An unmarked version of your revised paper without tracked changes. You should upload this as a separate file labeled 'Manuscript'.

If you would like to make changes to your financial disclosure, competing interests statement, or data availability statement, please make these updates within the submission form at the time of resubmission. Guidelines for resubmitting your figure files are available below the reviewer comments at the end of this letter.

As the corresponding author, your ORCID iD is verified in the submission system and will appear in the published article. PLOS supports the use of ORCID, and we encourage all coauthors to register for an ORCID iD and use it as well. Please encourage your coauthors to verify their ORCID iD within the submission system before final acceptance, as unverified ORCID iDs will not appear in the published article. Only the individual author can complete the verification step; PLOS staff cannot verify ORCID iDs on behalf of authors.

We look forward to receiving your revised manuscript.

Kind regards,

Chadi M. Saad-Roy

Academic Editor

PLOS Computational Biology

Benjamin Althouse

Section Editor

PLOS Computational Biology

**Reviewers' comments:**

Reviewer's Responses to Questions

**Comments to the Authors:**

**Please note here if the review is uploaded as an attachment.**

Reviewer #1: The authors have suitably addressed the comments. The work is an excellent contribution to applied literature on global sensitivity analysis. A few minor comments remain.

The authors have added units to Table 2 and clarified which quantities are rates and which are probabilities, which has greatly clarified the parameterization. However, $\eta_p$ is still described as “NHP disease death” rather than “NHP disease death rate” in table 2. Updating this would be more consistent with the other rate parameters in the table.

Are mosquito carrying capacities fixed throughout a simulation or change as host population sizes change? Although it is mentioned that K is set to twice the host size in each patch in Fig. 3 caption, does it mean they are fixed at twice the initial host population size in each patch?

Reviewer #2: I have no further comments.

**********

**Have the authors made all data and (if applicable) computational code underlying the findings in their manuscript fully available?**

The PLOS Data policy requires authors to make all data and code underlying the findings described in their manuscript fully available without restriction, with rare exception (please refer to the Data Availability Statement in the manuscript PDF file). The data and code should be provided as part of the manuscript or its supporting information, or deposited to a public repository. For example, in addition to summary statistics, the data points behind means, medians and variance measures should be available. If there are restrictions on publicly sharing data or code —e.g. participant privacy or use of data from a third party—those must be specified.

Reviewer #1: Yes

Reviewer #2: None

**********

PLOS authors have the option to publish the peer review history of their article (what does this mean?). If published, this will include your full peer review and any attached files.

If you choose “no”, your identity will remain anonymous but your review may still be made public.

**Do you want your identity to be public for this peer review?**  For information about this choice, including consent withdrawal, please see our Privacy Policy.

Reviewer #1: No

Reviewer #2: No

[NOTE: If reviewer comments were submitted as an attachment file, they will be attached to this email and accessible via the submission site. Please log into your account, locate the manuscript record, and check for the action link "View Attachments". If this link does not appear, there are no attachment files.]

**Figure resubmission:**

After uploading your figures to PLOS’s NAAS tool - https://ngplosjournals.pagemajik.ai/artanalysis, NAAS will process the files provided and display the results in the "Uploaded Files" section of the page as the processing is complete. If the uploaded figures meet our requirements (or NAAS is able to fix the files to meet our requirements), the figure will be marked as "fixed" above. If NAAS is unable to fix the files, a red "failed" label will appear above. When NAAS has confirmed that the figure files meet our requirements, please download the file via the download option, and include these NAAS processed figure files when submitting your revised manuscript.

**Reproducibility:**

To enhance the reproducibility of your results, we recommend that authors of applicable studies deposit laboratory protocols in protocols.io, where a protocol can be assigned its own identifier (DOI) such that it can be cited independently in the future. Additionally, PLOS ONE offers an option to publish peer-reviewed clinical study protocols. Read more information on sharing protocols at https://plos.org/protocols?utm_medium=editorial-email&utm_source=authorletters&utm_campaign=protocols

# Author comment: Author response to Decision Letter 3

**DOI:** 10.1371/journal.pcbi.1013952.r006

30 Sep 2026

Attachment Submitted filename: reply.pdf

# Editor report: Decision Letter 3

**DOI:** 10.1371/journal.pcbi.1013952.r007

**Authors:** Chadi Saad-Roy, Benjamin Althouse

14 Oct 2026

Dear Mr. Kornetzke,

We are pleased to inform you that your manuscript 'Global Sensitivity Analysis for Stochastic Models of Infectious Diseases' has been provisionally accepted for publication in PLOS Computational Biology.

Before your manuscript can be formally accepted you will need to complete some formatting changes, which you will receive in a follow up email. A member of our team will be in touch with a set of requests.

Please note that your manuscript will not be scheduled for publication until you have made the required changes, so a swift response is appreciated.

IMPORTANT: The editorial review process is now complete. PLOS will only permit corrections to spelling, formatting or significant scientific errors from this point onwards. Requests for major changes, or any which affect the scientific understanding of your work, will cause delays to the publication date of your manuscript.

Should you, your institution's press office or the journal office choose to press release your paper, you will automatically be opted out of early publication. We ask that you notify us now if you or your institution is planning to press release the article. All press must be co-ordinated with PLOS.

Thank you again for supporting Open Access publishing; we are looking forward to publishing your work in PLOS Computational Biology.

Best regards,

Chadi M. Saad-Roy

Academic Editor

PLOS Computational Biology

Benjamin Althouse

Section Editor

PLOS Computational Biology

***********************************************************

# Editor report: Acceptance letter

**DOI:** 10.1371/journal.pcbi.1013952.r008

**Authors:** Chadi Saad-Roy, Benjamin Althouse

PCOMPBIOL-D-26-00184R3

Global Sensitivity Analysis for Stochastic Models of Infectious Diseases

Dear Dr Kornetzke,

I am pleased to inform you that your manuscript has been formally accepted for publication in PLOS Computational Biology. Your manuscript is now with our production department and you will be notified of the publication date in due course.

The corresponding author will soon be receiving a typeset proof for review, to ensure errors have not been introduced during production. Please review the PDF proof of your manuscript carefully, as this is the last chance to correct any errors. Please note that major changes, or those which affect the scientific understanding of the work, will likely cause delays to the publication date of your manuscript.

Soon after your final files are uploaded, unless you have opted out, the early version of your manuscript will be published online. The date of the early version will be your article's publication date. The final article will be published to the same URL, and all versions of the paper will be accessible to readers.

For Research, Software, and Methods articles, you will receive an invoice from PLOS for your publication fee after your manuscript has reached the completed accept phase. If you receive an email requesting payment before acceptance or for any other service, this may be a phishing scheme. Learn how to identify phishing emails and protect your accounts at https://explore.plos.org/phishing.

Thank you again for supporting PLOS Computational Biology and open-access publishing. We are looking forward to publishing your work!

With kind regards,

Sharmila Kamatchi

PLOS Computational Biology | Carlyle House, Carlyle Road, Cambridge CB4 3DN | United Kingdom ploscompbiol@plos.org | Phone +44 (0) 1223-442824 | ploscompbiol.org | @PLOSCompBiol
